# Reducing Black–White Racial Differences on Intelligence Tests Used in Hiring for Public Safety Jobs

**DOI:** 10.3390/jintelligence11040062

**Published:** 2023-03-28

**Authors:** Harold W. Goldstein, Kenneth P. Yusko, Charles A. Scherbaum, Elliott C. Larson

**Affiliations:** 1 Department of Psychology, Baruch College, City University of New York, New York, NY 10016, USA; 2 Department of Psychology, University of Maryland, College Park, MD 20742, USA

**Keywords:** cognitive ability testing, group differences, adverse impact, public safety hiring, modern cognitive tests

## Abstract

This paper explores whether a diversity and inclusion strategy focused on using modern intelligence tests can assist public safety organizations in hiring a talented diverse workforce. Doing so may offer strategies for mitigating the issues of systematic racism with which these occupations have historically struggled. Past meta-analytic research shows that traditional forms of intelligence tests, which are often used in this sector, have not consistently demonstrated predictive validity but have negatively impacted Black candidates. As an alternative, we examine a modern intelligence test that consists of novel unfamiliar cognitive problems that test takers must solve without relying on their prior experience. Across six studies of varying public safety jobs (e.g., police, firefighter) in different organizations, we found a pattern of results that supports the criterion-related validity of the modern intelligence test. In addition to consistently predicting job performance and training success, the modern intelligence test also substantially mitigated the observed Black–White group differences. The implications of these findings are discussed in terms of how to alter the legacy of I/O psychology and human resource fields when it comes to our impact on facilitating employment opportunities for Black citizens, particularly in public safety positions.

## 1. Introduction

The year 2020 marked a year in which issues of social injustice and systematic racism moved to the forefront of the national discourse in the United States. To this end, the current article centers on a key flashpoint in this discussion, which is the role of the police when it comes to systematic racism against Black citizens. Headlines in 2020 regarding police brutality and unwarranted use of force against Black citizens were accompanied by a search for solutions that ranged from building trust with the public, recommitting to community-focused policing strategies, reengineering the culture of police organizations, and even defunding the police while putting in place alternative models of law enforcement. For those who study organizational science targeted at interventions leveraging human capital, the focus has predominately centered on delivering improved training-based strategies and solutions such as bias training, de-escalation techniques, and enhanced technical instruction such as appropriate use of deadly force (e.g., Correll et al. 2007; James 2018; President’s Task Force on 21st Century Policing 2015; Ruggs 2016). While these tactics can be effective, another approach that should be considered includes the hiring of talented diverse police officers, which has thus far received minimal attention (Donohue 2020; for exceptions, see Cascio et al. 2010; Hough 2016; Luse et al. 2021). In fact, the final report of a task force on policing for the 21st century commissioned under the Obama administration devotes less than a full page of a 116-page report to changes in how entry-level police officers are hired.

Given the incredibly challenging and complex nature of the police officer job, enhancing personnel selection systems to improve the talent level of police officers can be an effective and important strategy for meaningful police reform. In particular, it is essential to have police officers that use better judgment on the job and handle decisions effectively when under stress, qualities that might go a long way toward mitigating some of the horrific outcomes Black citizens have suffered when interacting with police. A focus on hiring individuals with strong competencies related to intelligence and decision-making can be part of a multifaceted approach to creating a public safety agency that better serves all communities (Hough 2016; Zabel et al. 2016). As it would happen, many in the field of personnel selection already believe that intelligence tests are the best tool we have for making hiring decisions (Murphy et al. 2003; Scherbaum et al. 2012; Sackett et al. 2022; Schmidt and Hunter 1998). Therefore, using them to select police officers is arguably an effective strategy that could help address the problems seen in policing today when it comes to racism. However, herein lies the quandary. First, while police and public safety jobs often use some form of intelligence test as part of their hiring process (Cochrane et al. 2003; Cox et al. 2018; Jacobs et al. 2011), research shows that traditional intelligence tests do not predict particularly well when it comes to police and public safety-type jobs (Hirsh et al. 1986; Salgado et al. 2003). Second, these traditional intelligence tests consistently produce racial differences that negatively impact Black candidates in comparison to White candidates (Hough et al. 2001). Thus, use of traditional intelligence tests hinders attempts to diversify the demographic makeup of the police workforce, which is another strategy for addressing systematic racism in that a racially diverse police force could arguably better relate to the diverse community that it serves (e.g., Morison 2017; Weitzer 2000).

To address this quandary, the current paper examines whether a modern intelligence test could be constructed for use in entry-level selection for police and other public safety positions that validly predicts performance while reducing racial differences so that a talented diverse workforce can be hired. These modern tests of intelligence focus on measuring the intelligence construct while promoting diversity by using newly emerging techniques from many areas of psychology, such as reducing the extent to which the test taker can rely on previous knowledge, thus creating a more equal playing field for those from different experiential and educational backgrounds (Larson et al. 2018; Scherbaum et al. 2015). The current study discusses the design of these modern intelligence tests and presents multiple studies conducted across a range of public safety jobs that demonstrate the viability of this approach for hiring a diverse talented workforce. The findings are discussed in terms of their implications for positively impacting police reform via personnel selection-focused strategies.

### 1.1. Modern Intelligence Tests

Traditional assessments of intelligence are widely used in personnel selection primarily because of the reported empirical support that they consistently and strongly predict job performance and training outcomes (Schmidt and Hunter 1998). While such tests are lauded in terms of predictive validity, they have also been disparaged for differential performance outcomes for racial/ethnic groups. For example, scores on general cognitive ability tests are stated to typically be about 1.0 standard deviation higher for Whites compared to Blacks (Hough et al. 2001; Roth et al. 2001). In many ways, this expected Black–White racial difference on intelligence tests is unfortunately viewed as an unavoidable truism of personnel psychology and has been regrettably embraced by I/O psychology and related human resource-focused fields (see McDaniel and Kepes 2014, for an example of this perspective). By serving as the primary fields that research the validity of intelligence tests in predicting job performance and playing a large role in the human resource decisions that encourage using these tests, the current legacy of I/O psychology and human resources includes putting in place tests that discriminate against Black individuals and, consequently, drive inequality in employment. 

At the core of this issue is the fact that I/O psychology and human resource fields have built their measurements of intelligence almost exclusively based on the psychometric approach (Goldstein et al. 2009; Larson et al. 2018; Scherbaum et al. 2012). Though this approach has its value, proponents of this perspective have argued that intelligence can be measured across a wide range of methodologies and measures and that the content of the test matters less than its ability to load onto a single factor of intelligence (Gottfredson 2002; Ree et al. 2015; Spearman 1927). This has led to an overreliance on knowledge-based measures (Schneider and Newman 2015), which have typically been shown to lead to large group score differences (e.g., Aguinis and Smith 2007; De Corte 1999; Goldstein et al. 2002; Ployhart and Holtz 2008; Sackett et al. 2001; Sternberg and Wagner 1993) and clearly limits the content domain being measured (e.g., Alfonso et al. 2005; Chen and Gardner 2012). 

While the I/O psychology and human resource fields have embraced the psychometric approach to intelligence, other scientific disciplines have taken a broader perspective and propose that the Black–White differences observed on tests depend on the manner in which intelligence is defined and measured (Fagan 2000; Fagan and Holland 2002; Mackintosh 1998; Sternberg 2006). Providing support for this notion are research findings that demonstrate the size of the racial difference varies depending on how intelligence is conceptualized (Fagan and Holland 2002, 2007) and which measure is used to capture the construct (Hough et al. 2001; Naglieri 2005; Wasserman and Becker 2000). Such findings bolster a thesis in the existing literature that conceptualizations of the construct and characteristics of the measurement device contribute to the size of the Black–White mean score differences observed and that alternative approaches to assessing intelligence may demonstrate validity while producing lower adverse impact against protected groups such as Black individuals (e.g., Edwards and Arthur 2007; Goldstein et al. 2009; Larson et al. 2018; Malda et al. 2010; Naglieri et al. 2005; Sternberg 2006; van de Vijver 1997). Given the implications of Black–White racial differences on high-stakes tests involving intelligence, specifically in terms of access to jobs and education (e.g., Sackett et al. 2001), additional research aimed at developing different modern approaches for measuring this construct with reduced Black–White racial differences is urgently needed.

Furthermore, exploring modern approaches to intelligence testing when it comes to hiring for police and public safety jobs makes even more sense given findings on traditional intelligence tests for these occupations. As previously noted, while forms of traditional intelligence tests are commonly used when hiring in public safety occupations (Cochrane et al. 2003; Cox et al. 2018; Jacobs et al. 2011), research has indicated that they do not consistently show strong predictive validity for these types of jobs. In a meta-analysis examining the predictive validity of tests of general mental ability (GMA) across varying occupations in Europe, Salgado et al. (2003) consistently found significantly lower results for the police occupation compared to other occupations when it came to predicting both learning outcomes from training and job performance outcomes. As noted by Salgado et al. (2003), these results were consistent with meta-analytic findings on intelligence tests and job performance by Hirsh et al. (1986) that were conducted on law enforcement jobs in the United States. However, it should be noted that the Salgado et al. (2003) findings, with a limited number of studies, were not consistent with the meta-analytic findings of Hirsh et al. (1986) that intelligence tests demonstrate validity when it comes to predicting training outcomes in law enforcement. Given that these traditional intelligence tests do not have a strong track record when it comes to predictive validity of job performance for police occupations and have somewhat inconsistent results when it comes to predicting training outcomes, exploring a different approach to measuring intelligence that may produce validity while also reducing the typical race differences associated with traditional approaches seems warranted.

One prescription for mitigating potential race differences is to identify aspects of the assessment approach or instrument that may be driving these differences and revise the tests accordingly. For example, several researchers have suggested that racial and ethnic group mean differences could be reduced by modifying certain elements of a traditional intelligence test to limit the extent to which test takers can rely on previously acquired information and knowledge (Fagan 2000; Malda et al. 2010; Sternberg 1981). These researchers view tests that allow for or require the use of previously acquired information and knowledge as contaminated. Furthermore, they reason that various racial and cultural groups have differential access and exposure to this information and knowledge which thus contributes to the group differences observed on such tests. In support of this rationale, research has shown that developing tests of general intelligence that reduce reliance on prior knowledge of language (e.g., Freedle and Kostin 1997), of relationships (such as quantitative reasoning) (e.g., Fagan and Holland 2002), and of how to complete tasks (e.g., Sternberg 1981) has resulted in decreased racial differences.

In fact, not reducing the extent to which test takers can rely on previously acquired information and knowledge is arguably a violation of a critical assumption of the psychometric approach to creating intelligence tests in that those taking the test must be ‘similarly situated’ (Jensen 1998; Ree and Carretta 2002). That is, they assert the content and the form of the test do not matter as long as the test takers perceive it in the same way. However, this may not be the case with typical traditional forms of intelligence tests because test takers may vary, perhaps by race, in terms of their previous knowledge about and familiarity with aspects of the test (Fagan 1992, 2000; Helms-Lorenz et al. 2003; Ortiz and Ochoa 2005). This lack of similar exposure to and familiarity with aspects of the test could arguably violate this assumption of the psychometric approach to intelligence and in addition may contribute to racial differences observed on the tests. To examine this issue, a number of researchers have investigated whether various ways of reducing differences in familiarity with aspects of the tests yields less racial group differences in performance. 

One approach used is to provide training in an attempt to equalize racial groups in terms of familiarity with aspects of the test. For example, Fagan and Holland (2002, 2007) conducted a series of studies comparing the performance of White and Black participants on vocabulary test items on which they possessed varying levels of prior knowledge and on vocabulary test items on which both groups were trained so that they arguably had equal familiarity with the items. As expected, the findings indicated that when participants were presented with items that relied on prior knowledge, White participants outperformed Black participants. However, when the same participants had an equal opportunity to learn new information through training, Black–White differences were greatly diminished. Other studies that have focused on training to equalize familiarity across groups include research that has shown that when racial groups are provided equal exposure to knowledge and information such as test-solving strategies, group differences in performance are reduced or disappear (e.g., Buttram 1975; Skuy et al. 2002; Sternberg et al. 2002).

Another approach used to equalize familiarity across racial groups, and one employed in the current studies, is to design tests with which neither racial group is familiar. That is, rather than training one group in order to equalize familiarity across groups, researchers have instead attempted to develop novel tests and procedures to which neither racial group has prior exposure. In fact, some researchers argue that intelligence is best measured using novel tasks that require advanced reasoning and information processing without relying on experience (Sternberg 1981). Even definitions of higher-level intelligence processes such as fluid intelligence focus on thinking logically and solving problems in novel situations, independent of acquired knowledge and familiar information (Carroll 1993; Cattell 1971; McGrew 2005).

Research has indeed provided some support that tests consisting of these novel stimuli can yield less discriminatory outcomes (Ortiz and Ochoa 2005; Valdés and Figueroa 1994). For instance, Sternberg found smaller racial differences when using novel, or what he refers to as ‘non-entrenched’ tasks (Sternberg 1981), as part of a battery meant to augment the Scholastic Aptitude Test (SAT) for his Rainbow Project (Sternberg 2006). Outtz and Newman (2009) found that tests measuring fluid intelligence (i.e., tests that focus on novel problem-solving), which racial groups should be equally unfamiliar with, tended to have smaller racial differences when compared with tests measuring crystallized intelligence (i.e., cognitive tests that focus on previously accumulated knowledge) with which racial groups could have differential exposure and familiarity. While some have questioned these findings, the positive results certainly argue for continued research on this strategy.

### 1.2. Current Studies 

Drawing from these previous positive findings, the present study focuses on a test of intelligence that uses novel cognitive tasks to reduce the reliance on previously acquired knowledge and information in the context of hiring for public safety jobs. In particular, we look at if this test can demonstrate predictive validity in hiring while significantly reducing the discriminatory Black–White differences that are typically observed in this sector. The test of intelligence used consists of reasoning items that require an individual to process, integrate, and manipulate information in order to identify trends, draw conclusions, and solve problems. Rather than the typical reasoning items found on many traditional intelligence tests (e.g., vocabulary, math equations, analogies), these items were designed to be novel in form so that test takers could not rely on prior knowledge to solve them. This was accomplished by reducing the use of previously learned language and quantitative skills, increasing the use of graphical stimuli and fake words, and having test takers perform novel ‘unentrenched’ tasks that they are not typically exposed to (e.g., an analogy is a task format that many are previously familiar with; however, novel mental puzzles that operate in an atypical manner are less familiar). For examples of similar approaches to item design, one can review the work undertaken by Fagan and Holland (2002, 2007) as well as Sternberg (2006). In summary, the items on this intelligence test were developed using a variety of approaches based on the principles outlined in modern intelligence research (e.g., Bosco et al. 2015; Fagan 1992, 2000; Flanagan et al. 2007; Helms-Lorenz et al. 2003; Higgins et al. 2007; Naglieri 2005; Ortiz and Ochoa 2005; Sternberg 1981, 2006). A sample item is included in the Appendix A. 

An initial version of the intelligence test consisted of a pool of 45 reasoning items that capture an individual’s ability to reason, problem solve, and make decisions on novel tasks and stimuli. Later versions of the test added a wider array of items to expand the pool to hundreds of items, along with parallel forms of many items. In terms of time allotment for completing the test, we budgeted one minute per item based on pilot studies that supported that most participants could complete an item in that time period. We conducted this purposively to reduce the extent to which the test involved time pressure, given such pressure has been thought to contribute to race/ethnicity differences observed on intelligence and cognitive tests. In terms of reliability, a recent sample of 3612 participants across multiple jobs and industries yielded a coefficient alpha of 0.79 for a short form of this intelligence test that consisted of 25 items. Research on the intelligence test generated strong foundations for the construct validity of the measure as a test of intelligence (e.g., using an employee sample of 196 participants from different work organizations, the intelligence test correlated with the Wonderlic intelligence test at 0.63 and with the Raven Progressive Matrices at 0.64 (Yusko et al. 2012), which aligns with typical research findings regarding the intercorrelation of intelligence tests (Daniel 2000). In terms of criterion-related validity evidence across 16 studies, various versions of this intelligence test have demonstrated a mean validity significant uncorrected correlation of 0.33 in predicting supervisor ratings of job performance across a wide range of jobs and industries (Yusko et al. 2012). This level of prediction fits well with typical findings from the field (i.e., Sackett et al. 2022). Finally, research on racial differences produced by this test typically show greatly reduced Black–White differences when compared to the 1.0 SD typically observed, usually ranging from a quarter to a half of a standard deviation (Yusko et al. 2012). 

Different versions of this intelligence test, as specifically described in each study that follows, were used in the current study. The study examines the predictive validity for multiple types of important criteria (e.g., job performance, learning outcomes) and Black–White racial differences in the intelligence test across a range of public safety jobs in order to determine the viability of using modern intelligence tests to help address systematic racism in hiring for these positions.

## 2. Methods and Results

### 2.1. Overview of Studies

The studies included in this paper, all of which are previously unpublished, come from a range of public safety organizations and jobs. Consistent with the focus of this article, some studies were conducted in police and law enforcement settings while others come from varying types of public safety organizations (e.g., fire departments). The benefit of this approach is it helps demonstrate the generalizability of the findings across the public safety context. We examined a diverse set of criteria measures across studies, including supervisor ratings and rankings of performance and learning outcomes (i.e., training success). The same intelligence test was employed throughout these studies though each version differed in the number of items on the test. Thus, the exact item makeup of the test differs from study to study, though each test sampled from a diverse set of item types built on the same set of principles described in the previous sections. In the following sections, we describe the sample, job and organization, the version of the test, the criterion measures, the relationship between the intelligence test and the criterion measures, and group score differences on the intelligence test. Summaries of the results of these studies can be found in Table 1 and Table 2. Each study was preceded by a structured job analysis process that identified information processing, decision-making, and problem-solving in novel and changing situations as critical capabilities that are needed from day one to perform the critical tasks of these jobs.

### 2.2. Study 1 Method and Results

This field study involved entry-level police officers in a mid-sized southern U.S. city. As part of a criterion-related validity study, data on the intelligence test were collected for 153 incumbents in the department. No demographic data were available for the participants in this particular study. A 24-item version of the intelligence test was used. The items were all novel learning application reasoning problems that required the candidate to learn relationships between a mixture of real and fake words and subsequently apply this learning to answer questions. Job performance ratings were collected from the incumbents’ Lieutenants around the same time as the intelligence test was administered. The performance measures included ratings on the critical performance dimensions identified in the job analysis. The composite representing the average of these ratings was used in the analyses.

Of the 153 incumbents, there were 112 for which we obtained both their intelligence test score and a performance rating. There was a statistically significant zero-order correlation between the intelligence test scores and the supervisor rating of job performance, *r* = .24, *p* < .01. The magnitude of this uncorrected correlation is approximately twice the size of the uncorrected meta-analytic correlations reported in the literature for police officer jobs (e.g., Hirsh et al. 1986; Salgado et al. 2003) and similar to the uncorrected correlation of intelligence tests with job performance ratings more generally (e.g., Bobko et al. 1999; Sackett et al. 2022).

### 2.3. Study 2 Method and Results

This field study involved entry-level police officers in a mid-sized midwestern U.S. city. As part of a criterion-related validity study, data on the intelligence test and job performance were collected for 158 incumbents in the department (n_Black Officers_ = 13; n_White Officers_ = 141; n_Native American Officer_ = 1; n_Asian Pacific Islander Officers_ = 2; n_Hispanic Officers_ = 1; n_Female Officers_ = 23; n_Male Officers_ = 135). A 53-item version of the intelligence test was used. The test consisted of 34 novel learning application reasoning problems similar to the items used in Study 1; the test also had 9 graphical reasoning problems that required the candidate to learn relationships between visual graphic images and apply this learning to answer questions; and lastly, the test also contained 10 novel mental puzzles that the candidate had to solve. Job performance ratings were collected from the incumbents’ Lieutenants around the same time as the intelligence test was administered. The performance measures included ratings on the critical performance dimensions identified in the job analysis. The composite representing the average of these ratings was used in the analyses.

There was a statistically significant zero-order correlation between the intelligence test scores and the supervisor rating of job performance, *r* = .25, *p* < .01. Again, the magnitude of this uncorrected correlation is approximately twice the size of the uncorrected meta-analytic correlations reported in the literature for police officer jobs (e.g., Hirsh et al. 1986; Salgado et al. 2003) and similar to the uncorrected correlation of intelligence tests with job performance ratings more generally (e.g., Bobko et al. 1999; Sackett et al. 2022). Furthermore, we analyzed the group score differences and found a *d* value of .48, favoring White incumbents. Although the sample size of Black officers is small, the magnitude of the difference is half of the typical score difference reported in the literature (e.g., Roth et al. 2003). In the following studies, we present group score differences with larger sample sizes of candidates and incumbents that replicate these findings.

### 2.4. Study 3 Method and Results

This field study involved deputy sheriffs in a mid-sized mid-Atlantic U.S. city. Data on the intelligence test were collected for 124 incumbents in the department (n_Black Deputy Sheriffs_ = 62; n_White Deputy Sheriffs_ = 47; n_Asian Pacific Islander Officers_ = 6; n_Hispanic Officers_ = 8; n_Female Officers_ = 26; n_Male Officers_ = 97; no demographic data were provided on 1 incumbent). A 26-item version of the intelligence test was used. The test consisted of all novel learning application reasoning problems similar to the items used in Study 1. Additionally, intelligence test scores were collected from candidates applying to this job (n_Black Candidates_ = 935; n_White Candidates_ = 852; no additional demographic data were available for these candidates). The test scores from these candidates were used to examine group score differences. Job performance ratings were collected from the incumbents’ Lieutenants around the same time as the intelligence test was administered. The performance measures included ratings on the critical performance dimensions identified in the job analysis. The composite representing the average of these ratings was used in the analyses. As part of an additional study, ratings on learning outcomes (i.e., training success) were collected from training academy personnel for 39 of the incumbent deputy sheriffs (no demographic data were available for this sample).

Of the 124 incumbents, there were 83 for which we obtained both their intelligence test score and a performance rating. For these individuals, there was a statistically significant zero-order correlation between the intelligence test scores and the supervisor ratings of job performance, *r* = .27, *p* < .05. As was the case for Studies 1 and 2, the magnitude of this validity coefficient is approximately twice the size of the value reported in the literature for police officer jobs and in line with the value for other jobs reported in the literature. For the training academy ratings, the uncorrected zero-order correlation was statistically significant, *r* = 0.49, *p* < .01. This value exceeds the uncorrected meta-analytic correlations that have been reported in the literature for police officer training success (e.g., Hirsh et al. 1986; Salgado et al. 2003). For the incumbents, we found a *d* value of .35, favoring White incumbents. For the candidates, we found a *d* value of .41 favoring White candidates. Consistent with Study 2, the size of the group differences on a modern intelligence test is less than half of what is reported in the literature for traditional intelligence tests, but the validity coefficients are larger than what has been reported in the literature.

### 2.5. Study 4 Method and Results

While Studies 1 through 3 demonstrated the criterion-related validity and group score differences for the intelligence test in law enforcement settings, Studies 4 and 5 present data from firefighters to examine the generalizability of these findings to other public safety jobs. Common to law enforcement and firefighting jobs are concerns about how the entry-level testing serves as a barrier to hiring diverse employees.

Study 4 was a field study involving entry-level firefighters in a mid-sized midwestern U.S. city. As part of a criterion-related validity study, data on the intelligence test and job performance were collected for 153 incumbents in the department. No demographic data were available on these incumbents. A 22-item version of the intelligence test was used. The test consisted of 9 novel learning application reasoning problems similar to the items used in Study 1; the test also had 6 graphical reasoning problems that required the candidate to learn relationships between visual graphic images and apply this learning to answer questions; and lastly, the test also contained 7 novel mental puzzles that the candidate had to solve. Additionally, intelligence test scores were collected from candidates applying to this job (n_Black Candidates_ = 162; n_White Candidates_ = 773; n_Native American Officer_ = 6; n_Asian Pacific Islander Officers_ = 8; n_Hispanic Officers_ = 13; n_Female Officers_ = 76; n_Male Officers_ = 933; please note that race/ethnicity data were not reported for some individuals). The test scores from these candidates were used to examine group score differences. Job performance ratings were collected from the incumbents’ battalion chiefs around the same time as the intelligence test was administered. The performance measure was a ranking of the incumbents on overall performance.

There was a statistically significant zero-order correlation between the intelligence test scores and the supervisor ranking of job performance, *r* = .25, *p* < .01. In the candidate sample, we found a *d* value of .46, favoring White candidates. Thus, in a separate public safety job, we observe a similar pattern of statistically significant correlations between scores from a modern intelligence test and job performance, but much smaller score differences between race groups.

### 2.6. Study 5 Method and Results

As part of a field study, we collected data for firefighter candidates over a five-year period in a mid-sized southern U.S. city. Through this process, intelligence tests were collected from candidates using a 20-item version of the intelligence test (nBlack Candidates = 1227; nWhite Candidates = 1188; no additional demographic data were available). The test consisted of all novel learning application reasoning problems similar to the items used in Study 1. Learning outcomes were available on 123 of these candidates in the form of ratings of training success from training academy personnel (n_Black Candidates_ = 36; n_White Candidates_ = 77; n_Other Candidates_ = 14; n_Female Officers_ = 8; n_Male Officers_ = 111; some demographic gender data were missing).

There was a statistically significant zero-order correlation between the intelligence test scores and the training success, *r* = .38, *p* < .001. Across all candidates, we found a *d* value of .43, favoring White candidates. Similar to Study 4, we find that the modern intelligence test produces smaller score differences among firefighter candidates and is related to learning outcomes.

### 2.7. Study 6 Method and Results

This field study involved law enforcement analysts from a federal agency. As part of a criterion-related validity study, data on the intelligence test were collected for 446 incumbents in the agency (n_Black_ = 36; n_White_ = 338; additional demographic data were not available). A 25-item version of the intelligence test was used. The test consisted of 9 novel learning application reasoning problems similar to the items used in Study 1; the test also had 6 graphical reasoning problems that required the candidate to learn relationships between visual graphic images and apply this learning to answer questions; and lastly, the test also contained 10 novel mental puzzles that the candidate had to solve. Overall job performance ratings were collected from the incumbents’ supervisor around the same time as the intelligence test was administered.

Of the 446 incumbents, there were 299 for which we obtained both their intelligence test score and a performance rating. There was a statistically significant zero-order correlation between the intelligence test scores and the supervisor rating of job performance, *r* = .25, *p* < .001. We found a *d* value of .38, favoring White incumbents. Study 6 replicates the pattern observed in other law enforcement jobs of evidence of validity for the modern intelligence test and smaller score differences between race groups.

### 2.8. Meta-Analytic Estimate of Group Score Differences

When computing the group score differences in our samples, we consistently found the *d* values to be below .50. However, the confidence intervals across our samples varied considerably due to some studies having small sample sizes, particularly for Black incumbents/candidates. To more accurately estimate the *d* value and its associated confidence interval, we conducted a bare-bones meta-analysis across the six samples (Hunter and Schmidt 2004). We found that the sample-size weighted meta-analytic estimate was .42. The 95% confidence interval (.40, .45) indicates that our meta-analytic estimated *d* value is half the typical group score difference found on intelligence tests (Hough et al. 2001; Roth et al. 2001).

## 3. Discussion

When the Civil Rights Act was passed in 1964 in the United States, the focus was on prohibiting discrimination based on race, color, religion, sex, and national origin. While the act protected all these groups, most view the way Black citizens were treated in the United States as the impetus for this legislation (Aiken et al. 2013). The inclusion of Title VII in the act was a crucial component that recognized that without providing an avenue for economic equality by prohibiting discrimination when it came to hiring in work organizations, meaningful change in the quality of life for Black citizens would be difficult. However, over fifty years later, the United States is still struggling with issues of systematic racism and racial equality in work organizations (Lindsey et al. 2013). Particularly pertinent to the fields of I/O psychology and human resources is our continued legacy of implementing traditional psychometric intelligence tests into personnel selection systems that are detrimental to the hiring of Black candidates for jobs.

With this in mind, the current paper focused on whether a modern test of intelligence could be used to facilitate hiring of Black candidates into public safety jobs, an occupation fraught with charges of racist behavior and unnecessary use of force against Black citizens. The focal question was whether such tests would demonstrate predictive validity while mitigating the typical Black–White differences observed on traditional tests of intelligence. If successful, this could greatly enhance the ability of public safety organizations to hire a talented diverse workforce and still measure intelligence in the selection process. Such an approach can, over time and along with other targeted interventions, help improve diversity and reduce aspects of systematic racism associated with police and other public safety jobs (Chatterjee 2016). While the current study focused on United States issues and samples in examining the use of a modern test of intelligence, the findings should be of interest to other countries where issues of racial and cultural differences occur. In addition, a finding that intelligence tests that reduce the amount of prior knowledge needed to complete them could be of interest to any countries or groups where unequal learning opportunities exist.

In contrast to prior research that shows traditional forms of intelligence tests are not particularly valid in public safety occupations (e.g., Hirsh et al. 1986; Salgado et al. 2003), our pattern of results showed that the modern intelligence test used in our research consistently demonstrated predictive validity for both job performance and learning outcomes across a variety of public safety jobs. Across six independent studies of different public safety positions, the modern intelligence test consistently showed validity in predicting supervisor ratings of job performance (i.e., significant uncorrected correlations ranging from 0.24 to 0.27). In addition, two studies provided support that the modern intelligence test demonstrated strong relationships with learning outcomes related to training academy success in the public safety context (i.e., significant uncorrected correlations ranging from 0.38 to 0.49). In comparison to findings in the research literature, the modern intelligence test employed in the current study demonstrably outperformed traditional intelligence tests based on past meta-analyses findings in public safety settings (e.g., Salgado et al. 2003, report an uncorrected validity of 0.12 for job performance ratings and 0.13 for training success, while Hirsh et al. 1986, report an uncorrected validity of 0.08 for job performance ratings and 0.30 for training success).

This pattern of results supports the use of modern intelligence tests to help select talented individuals for public safety occupations, but the critical remaining question is whether such an approach will help increase diversity in these jobs. The results across our studies consistently demonstrated dramatically reduced Black–White differences on the modern intelligence test compared to what is found with typical traditional tests of intelligence. The *d* for the multiple studies ranges from 0.35 to 0.48 in terms of White individuals outperforming Black individuals on the test, which falls well below the d of 1.00 typically associated with intelligence tests (Hough et al. 2001; Roth et al. 2003). Thus, for all the public safety jobs studied, whether examining current employees or job candidates, the modern intelligence test produced racial differences of less than half the size found with traditional intelligence tests. This is a meaningful reduction that can yield positive outcomes in terms of hiring a more diverse workforce for public safety jobs.

Thus, while previous research on intelligence has historically stated that predictive validity and racial group differences are mutually exclusive (Sackett et al. 2001; McDaniel and Kepes 2014), the results of the current study demonstrate that this may not always be the case and that new modern approaches to the design of intelligence tests could support both goals concurrently. Based on these findings as well as those of others (e.g., Fagan and Holland 2007; Sternberg 1981, 2006), it could be argued that the traditional approach to measuring intelligence commonly used in employment settings, including public safety, needs to be reconsidered. The findings suggest that we need to critically evaluate current hiring methods and seriously consider alternative ways of measuring intelligence, including using techniques involving novel information and tasks that reduce the extent to which the test taker can rely on prior knowledge and experience. It is interesting to note that other tests of intelligence, particularly some that are focused on measuring fluid intelligence, have previously attempted to reduce racial and cultural differences but have been largely unsuccessful. For instance, the well-known Raven’s Progressive Matrices test was designed to mitigate racial and cultural differences by using graphical stimuli to reduce the verbal load of the assessment; however, the racial and cultural differences did not diminish (Raven 2000; Rindermann 2013). Some possible reasons why the modern test in the current study produced a different result are that the Raven’s Progressive Matrices did not use a wide range of item types as recommended by Jensen (1998) and in addition, it is possible that as more people were exposed to the Raven’s Progressive Matrices, the test was no longer novel in their eyes (i.e., test takers did have prior knowledge and experience with the assessment). Future research needs to look closely at these issues and the specific test design features of the modern intelligence tests to better understand how they reduce reliance on prior knowledge and experience to understand their impact on measuring the intelligence construct and the implications for racial differences (e.g., Agnello et al. 2015). While in the current study, a clear pattern between item type and resulting size of the racial difference did not emerge, future research should continue to delve deeper into this question. Such research can help science better understand the potential contaminating features of testing approaches and reduce their negative impact on the fairness of our tests.

Of course, this study is not without limitations. First, in a few of the incumbent samples, the number of Black incumbents is small, and the score differences may be unstable. However, the patterns observed in the smaller sample are replicated in the larger samples, alleviating this concern. Another limitation of our research is that we were not able to examine differential validity between White and Black test takers as race information was not available for the job incumbents in several of our studies. For the studies in which race information was available for the job incumbents, the sample sizes for the Black incumbents were very small. Consistent with other scholars, we encourage future research to examine differential validity with modern cognitive ability tests (e.g., Berry 2015). In addition, while we present solid evidence of criterion-related validity for this modern intelligence measure, future research should continue to focus on the psychometric properties of this measure, including evidence of structural validity and discriminant validity.

In addition, for some of the samples, we have limited information regarding in-depth demographic information (e.g., information on the gender and age of the sample). To truly understand demographic subgroup differences, future samples should be studied that have this more detailed demographic information. Additionally, our job performance criteria were limited to overall performance ratings or composites of dimensions of performance. We were unable to examine the relationships with specific dimensions of performance on these jobs. This is an important direction for future research to study the relationships with job performance dimensions that may vary in their cognitive orientation. In addition, this study did not include a conventional intelligence test to allow a direct comparison between the testing approaches. Future research should be conducted with both testing strategies so a direct comparison can be made. Such future research will also help add a deeper exploration of the construct validity of the modern measures of intelligence.

## 4. Conclusions

To conclude, our aim was to investigate a hiring strategy that could be used to make a contribution when it increases diversity and inclusion in work organizations. The focus of the article was on public safety organizations, which have historically struggled with systematic racism when it comes to interactions with the public and efforts to diversify their workforce. Some have called on the field of I/O psychology to engage in modern research on intelligence and to move away from the sole reliance on psychometric perspectives and their legacy of decreasing the employment opportunity of Black individuals (e.g., Scherbaum et al. 2012). The initial findings of doing so are encouraging, as seen in the results of this study that demonstrate the viability of using modern intelligence tests as a diversity and inclusion strategy, even in difficult settings such as public safety occupations. To change our legacy with regard to systematic racism, we need to further heed the call and strongly pursue with the utmost urgency streams of research such as this and quickly leverage the findings to put into practice the mechanisms needed to drive real change in work organizations.

## Figures and Tables

**Table 1:** Criterion-related validity in public safety jobs.

Study	Position	Sample	Criterion	# of Items	Alpha	Criterion-Related Validity
1	Entry-level police officer	Incumbents	Supervisor rating of job performance	24	0.82	*r* = .24, *p* < .01, N = 112
2	Entry-level police officer	Incumbents	Supervisor rating of job performance	53	0.80	*r* = .25, *p* < .01, N = 158
3	Deputy sheriff	Incumbents	Supervisor rating of job performance	26	0.82	*r* = .27, *p* < .05, N = 83
			Ratings of training success	26	0.82	*r* = .49, *p* < .01, N = 39
4	Entry-level firefighter	Incumbents	Supervisor ranking of employees	22	0.93	*r* = .25, *p* < .01, N = 153
5	Entry-level firefighter	Candidates	Ratings of training success	20	0.82	*r* = .38, *p* < .001, N = 123
6	Law enforcement analyst	Incumbents	Supervisor rating of job performance	25	0.72	*r* = .25, *p* < .001, N = 299

**Table 2:** Group score differences in public safety jobs.

Study	Position	Sample	Group Score Differences
2	Entry-level oolice officer	Incumbents	*d* = .48 (95% CI: −.09, 1.05) N_w_ = 141, N_b_ = 13
3	Deputy sheriff	Incumbents	*d* = .35 (95% CI: −.03, .73) N_w_ = 47, N_b_ = 62
		Candidates	*d* = .41 (95% CI: .32, .50) N_w_ = 852, N_b_ = 935
4	Entry-level firefighter	Candidates	*d* = .46 (95% CI: .36, .56) N_w_ = 773, N_b_ = 162
5	Entry-level firefighter	Candidates	*d* = .43 (95% CI: .35, .51) N_w_ = 1188, N_b_ = 1227
6	Law enforcement analyst	Incumbents	*d* = .38 (95% CI: .04, .72) N_w_ = 338, N_b_ = 36
Meta-analytic estimate (*k* = 6)			*d* = .42 (95% CI: .40, .45) N_w_ = 3354, N_b_ = 2420

**Figure A1:**
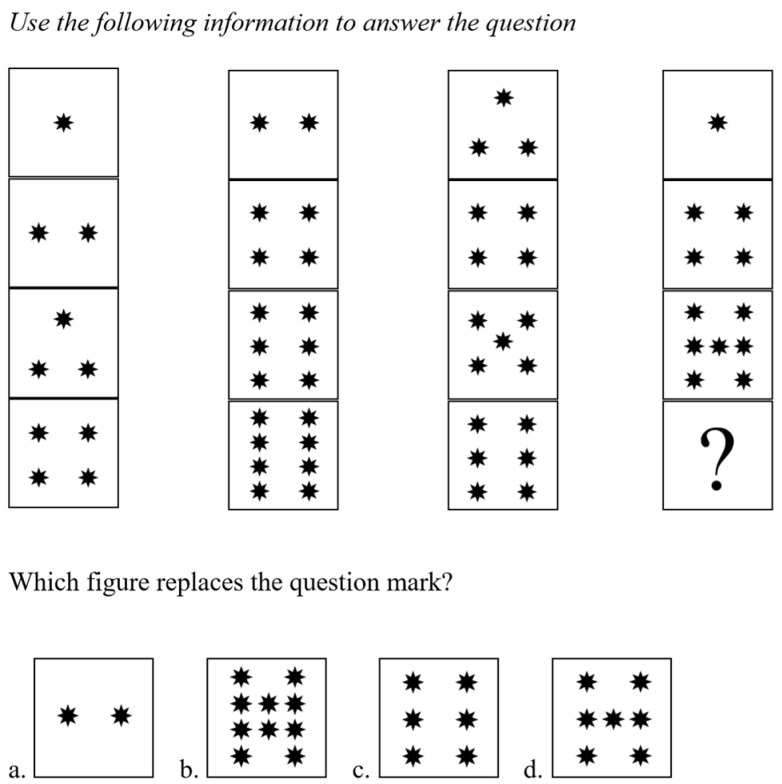
Sample item and answer. Instructions: This test measures your ability to solve problems and reason effectively. Read each question carefully and mark the single best answer. Please note: Option B is the correct answer based on the pattern of the figures.

## Data Availability

Due to ethical, legal, and privacy issues, these data are not publicly available and cannot be shared.
